# Correction: Yu et al. Non-Cationic RGD-Containing Protein Nanocarrier for Tumor-Targeted siRNA Delivery. *Pharmaceutics* 2021, *13*, 2182

**DOI:** 10.3390/pharmaceutics17111462

**Published:** 2025-11-13

**Authors:** Xiaolin Yu, Lu Xue, Jing Zhao, Shuhua Zhao, Daqing Wu, Hong Yan Liu

**Affiliations:** 1 Georgia Cancer Center, Augusta University, Augusta, GA 30912, USA; 2 Department of Pediatrics Hematology, The First Hospital of Jilin University, Changchun 130021, China; 3 Department of Gynecology and Obstetrics, The Second Hospital of Jilin University, Changchun 130041, China; 4 Center for Cancer Research and Therapeutic Development, Clark Atlanta University, Atlanta, GA 30314, USA; 5 Dotquant LLC, Seattle, WA 98195, USA

## Affiliation Update

We updated affiliation 5 from “Dotquant LLC, CoMotion Labs at University of Washington, Seattle, WA 98195, USA” to “Dotquant LLC, Seattle, WA 98195, USA”, as CoMotion Labs at University of Washington is a start-up incubator.

## Author’s Email Update

We have updated Prof. Hong Yan Liu’s email address from hongyl6@uw.edu to HOLIU@augusta.edu, as she is now an adjunct faculty of Augusta University.

## Funding Update

The data from this publication was developed at Augusta University, and the animal work was performed under Augusta University’s animal protocol. NIH (NIBIB) R21EB026564-01A supported the related work but no data in this publication. For clarification, the correct funding information appears below:

This work was supported by the start-up funding from Augusta University (to H.Y.L.) but not by external funding.

## Error in Figure

In the original publication [1], there was a mistake in Figure 8B. We inadvertently assembled tissue HE images from our previous publication [2]. We promptly reviewed the original data and confirmed that there was an oversight when assembling the images. The HE histology results of the two studies are similar: there are no differences between the controls and treatment groups, and this contributed to the insertion error. The correct version of Figure 8B appears below. The authors state that the scientific conclusions are unaffected. This correction was approved by the Academic Editor. The original publication has also been updated.

**Figure 8:**
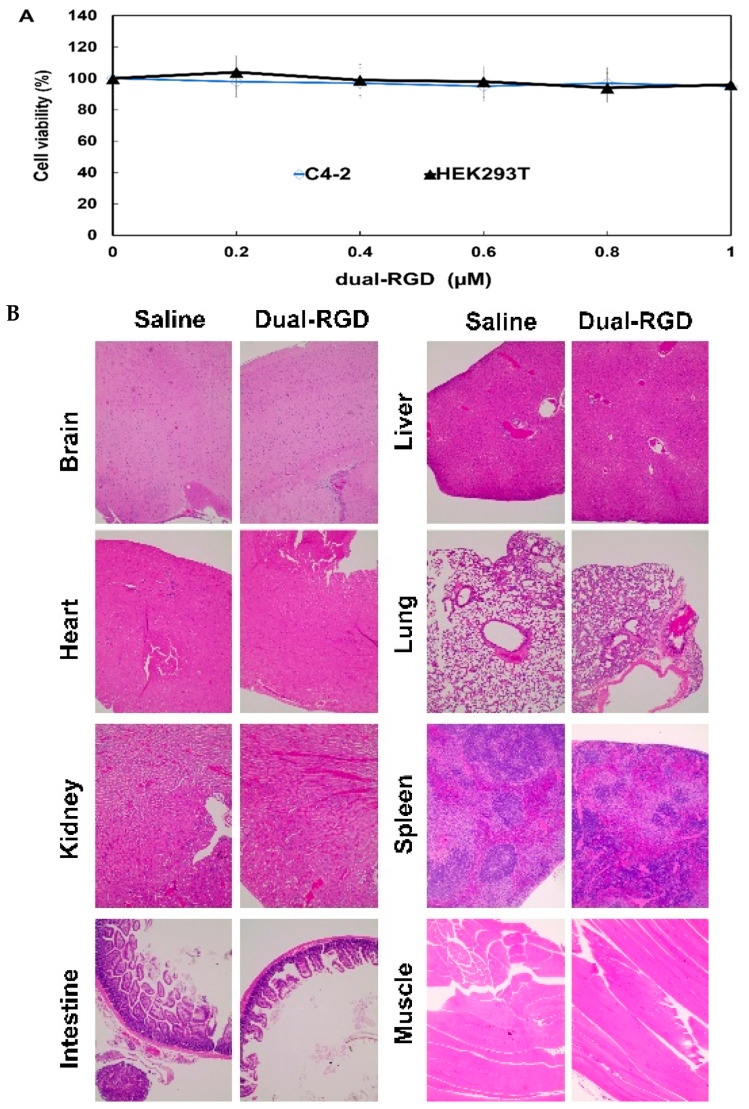
Assessment of toxicity. (**A**) Cell viability assay. Integrin α_v_β_3_-negative C4-2 and HEK293 cells were treated with the varying concentrations of dual-RGD for 72 h. CCK-8 reagent was added into each well for 4 h. Absorbance was measured at 450 nm. (**B**) Histology examination of dual-RGD on major normal organs. Athymic mice were treated with dual-RGD or saline for four weeks. After treatment, the major organs were fixed and stained with H&E. Images were captured under 20× magnification microscopy.
