# Supplementary material for: Non-Cationic RGD-Containing Protein Nanocarrier for Tumor-Targeted siRNA Delivery
Source: Pharmaceutics. 2021 Dec 17;13(12):2182. doi: 10.3390/pharmaceutics13122182 (PMC8703291; doi:10.3390/pharmaceutics13122182)
Supplement: Supplementary file 1 [file pharmaceutics-13-02182-s001.zip › pharmaceutics-1450762-supplementary.pdf]

pharmaceutics

Supplementary Materials: Non-Cationic RGD-Containing Protein Nanocarrier for Tumor-Targeted siRNA Delivery

Xiaolin Yu, Lu Xue, Jing Zhao, Shuhua Zhao, Daqing Wu and Hong Yan Liu

A

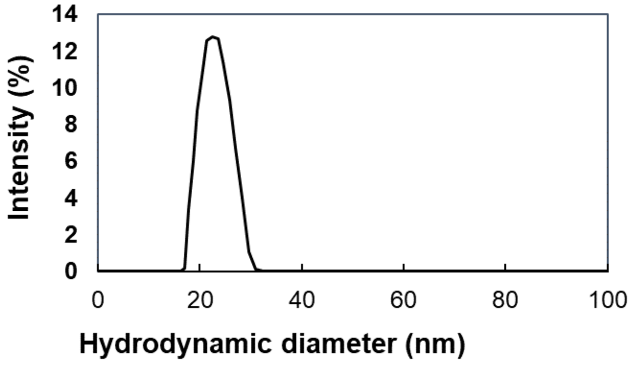

B

	Zeta Potential (mv)	Mobility (µmcm/Vs)	Conductivity (mS/cm)
Test 1	-7.87	-0.617	38.1
Test 2	-7.83	-0.6136	38.9
Test 3	-7.85	-0.6125	38.7
Mean	-7.85	-0.614	38.56
STD	0.02	0.002	0.41

Figure S1. Detection of hydrodynamic diameter and zeta potential by Zetasizer. Monomer dual-RGD in PBS buffer were measured at 25℃. The hydro-dynamic diameter is about 23.5nm shown in (**A**) and zeta potential is about -7.85mv shown in (**B**).

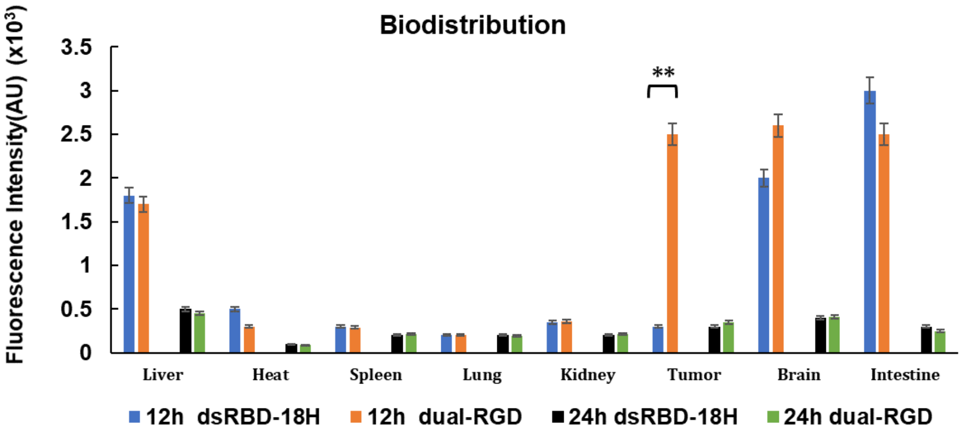

Figure S2. Ex vivo evaluation of biodistribution. **(A)** Athymic mice were injected with MDA-MB-231 cells (5 x10^6) mixed with Matrigel (v/v 1:1) subcutaneously. After 4 weeks, tumor-bearing mice were tail-vein injected with 100µl of Cy5-EGFR siRNA/dual-RGD complex (5 nmoles) or equal moles of Cy5-siRNA/dsRBD-18His complex. At time of 12h and 24h, major organs are removed and homogenized in buffer (10mM Tris pH7.4 and 0.5% Triton X-100) with mortar and pestle at a ratio of 100mg of tissue per ml buffer. 100µl of tissue homogenate was loaded to a 96-well plate. The plate was measured by Tecan Infinite F200 Pro Microplate Reader. The results are the mean ± SEM (N=3). **P<0.01.

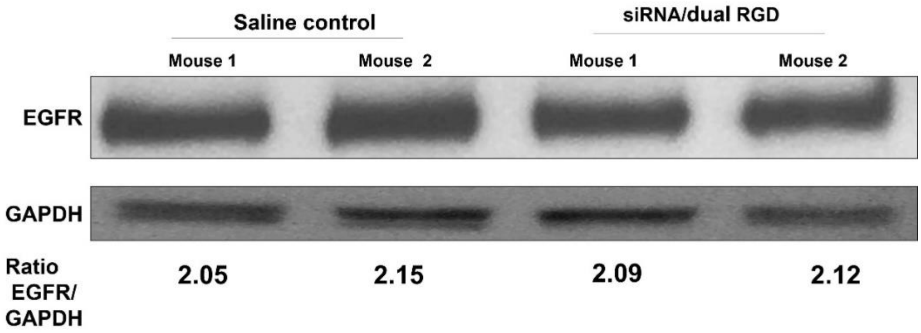

Figure S3. Evaluation of off-target gene silencing in brain. After treatment of saline or siRNA/dual RGD (5nmles) twice a week for 4 weeks. EGFR silencing in brain tissues was detected by Western blot. There is no significant difference of EGFR expression between saline treated or siRNA/dual RGD treated mouse brains. This result indicates dual-RGD did not induce siRNA endocytosis/gene knockdown in brain.
